# Sex Differences in Disease Activity Measures in Axial Spondyloarthritis and Their Association with Concomitant Fibromyalgia: A Retrospective Cross-Sectional Analysis of a Saudi Cohort

**DOI:** 10.3390/jcm15145602

**Published:** 2026-07-17

**Authors:** Mohamed Bedaiwi, Aos Aboabat, Ibrahim Almaghlouth, Tharaa S. Alhowaish, Haya M. Almalag, Eman Alqurtas

**Affiliations:** 1 Rheumatology Unit, Department of Medicine, College of Medicine, King Saud University, Riyadh 11461, Saudi Arabia; 2 Department of Clinical Pharmacy, College of Pharmacy, King Saud University, Riyadh 11149, Saudi Arabia

**Keywords:** axial spondyloarthritis, sex differences, fibromyalgia, obesity, body mass index, disease activity, regression analysis

## Abstract

**Background/Objectives:** Axial spondyloarthritis (axSpA) was long considered a male disease, yet women often report higher disease activity; whether this reflects sex differences or comorbidity is unclear. In an under-studied Saudi cohort, we examined sex differences in disease activity and function and associations with fibromyalgia and body mass index (BMI). **Methods:** A retrospective cross-sectional analysis of de-identified baseline data included 160 adults who met the Assessment of SpondyloArthritis International Society (ASAS) criteria at a single center. Between-sex comparisons used Benjamini–Hochberg correction; sequential multivariable regression evaluated attenuation of the sex–disease activity association after covariate adjustment. **Results:** Of 160 patients, 56 (35%) were women. Women more often had non-radiographic disease, higher BMI, fibromyalgia, and elevated erythrocyte sedimentation rate (ESR) (all *q* < 0.05), and scored higher on the symptom-based Bath Ankylosing Spondylitis Disease Activity Index (BASDAI; median 5.65 [IQR 4.67–6.60] vs. 5.15 [4.30–5.72]; *p* = 0.007, *q* = 0.044). The Bath Ankylosing Spondylitis Functional Index (BASFI) was borderline (*q* = 0.052), and C-reactive protein (CRP)-anchored Ankylosing Spondylitis Disease Activity Score (ASDAS-CRP) did not differ significantly (median 3.70 [IQR 3.00–3.90] vs. 3.50 [3.00–3.80]; *p* = 0.099, *q* = 0.148). The female BASDAI difference was attenuated after adjustment for concomitant fibromyalgia. Higher ESR was partly attenuated after adjustment for BMI, whereas CRP did not differ by sex. In the analysis restricted to participants without fibromyalgia, between-sex estimates for BASDAI, BASFI, and ASDAS-CRP were smaller and imprecise. **Conclusions:** The attenuation of the female-sex association with BASDAI after adjustment for concomitant fibromyalgia should be interpreted as statistical attenuation only. Because fibromyalgia and BASDAI share symptom domains, particularly pain and fatigue, this attenuation may partly reflect overlapping symptom content and possible classification circularity rather than an explanatory or causal relationship.

## 1. Introduction

Axial spondyloarthritis (axSpA) has long been considered a disease of men. The male preponderance of radiographic sacroiliitis and the strong association with HLA-B27 reinforced that view, and women were under-recognized and diagnosed later as a result [1,2]—a delay also documented in Saudi Arabia and the wider region [3,4]. However, this picture has changed. The Assessment of SpondyloArthritis International Society (ASAS) classification criteria [5] and the growing recognition of non-radiographic axSpA, which is as common in women as in men, have renewed interest in how the disease differs between the sexes [6].

Across cohorts, women with axSpA tend to report higher disease activity than men on patient-reported composite indices, such as the Bath Ankylosing Spondylitis Disease Activity Index (BASDAI). Measures incorporating an inflammatory biomarker, such as the Ankylosing Spondylitis Disease Activity Score using C-reactive protein (CRP; ASDAS-CRP), show little sex difference. A systematic review and meta-analysis captured this split—a clinically meaningful sex difference in BASDAI but none in ASDAS-CRP [7]. The meaning of the split is unsettled. This may reflect a genuine sex difference in disease expression or arise from factors that differ between men and women and increase patient-reported scores without reflecting axial inflammation.

Sex differences in reported disease activity are likely multifactorial and may reflect biological factors, radiographic phenotype, diagnostic delay, treatment exposure, comorbidities, psychosocial circumstances, pain perception and reporting, central sensitization, and cultural context. Among these factors, concomitant fibromyalgia and body mass index (BMI) are clinically relevant because both may influence patient-reported disease activity, physical function, and inflammatory markers. Fibromyalgia is more common in women and frequently co-occurs with axSpA [8,9], and it may increase pain- and fatigue-weighted BASDAI scores [10,11]. Higher BMI has also been associated with increased inflammatory markers and greater reported disease activity [12,13]. However, these factors should be considered possible contributors rather than exclusive explanations for observed sex differences. Data from Middle Eastern and Gulf populations remain scarce, particularly in settings where the prevalence of HLA-B27 [14,15], obesity, healthcare access, and sociocultural influences may differ from those in previously studied cohorts.

Therefore, we examined sex differences in disease activity measures and physical function in a single-center Saudi axSpA cohort. We assessed the possible contribution of concomitant fibromyalgia and BMI to these observed differences without assuming that they were the sole or exclusive explanation. The primary analysis focused on the association between sex and BASDAI and on the extent to which the female-sex coefficient was attenuated after sequential adjustment for clinical covariates, including BMI and fibromyalgia. We also compared the Bath Ankylosing Spondylitis Functional Index (BASFI), ASDAS-CRP, CRP, and the erythrocyte sedimentation rate (ESR) between women and men.

## 2. Materials and Methods

### 2.1. Study Design and Setting

The primary analysis was a retrospective cross-sectional analysis of de-identified baseline registry data; twelve-month follow-up outcomes were examined separately as an exploratory retrospective longitudinal component and are reported in the Supplementary Materials. Data were obtained from an ongoing single-center institutional axSpA registry at King Saud University Medical City, Riyadh, Saudi Arabia. The institutional registry received original ethics approval on 3 May 2020, valid to 3 May 2021 (protocol E-20-4626; Ref. No. 20/0223/IRB). Patients with baseline assessments between January 2021 and April 2025 were included in the primary cross-sectional analysis, and twelve-month follow-up assessments were completed by April 2026. During this period, patients were assessed and their clinical information was recorded as part of routine rheumatology care, with no prospective research-specific recruitment, intervention, study-specific visit, additional investigation, questionnaire, or alteration of clinical management for this manuscript. The most recent renewal available to the authors is designated Renewal No. 5 and was approved on 15 June 2026, valid to 15 June 2027 (Ref. No. 26/0464/IRB). The Renewal No. 5 document refers to the same ongoing registry protocol rather than to a new study-specific retrospective approval. Following this renewal, the final de-identified analytic extract used for the revised manuscript was generated, verified, and locked on 26 June 2026. No formal sample size calculation was performed; all eligible registry patients were included. The reporting of this study follows the STROBE recommendations for observational studies [16].

### 2.2. Participants

Eligible patients were adults (≥18 years) who fulfilled the Assessment of SpondyloArthritis International Society (ASAS) classification criteria for axSpA. Axial spondyloarthritis was diagnosed clinically by a rheumatologist, with ASAS criteria used for classification. Radiographic axSpA was defined by definite radiographic sacroiliitis according to the modified New York criteria, based on routine pelvic radiograph reports and rheumatology documentation; no independent central rereading was performed. Patients without definite radiographic sacroiliitis who fulfilled the ASAS classification criteria were classified as having non-radiographic axSpA. Sacroiliac-joint magnetic resonance imaging (MRI) was obtained when clinically indicated and could support classification of non-radiographic axSpA in patients without definite radiographic sacroiliitis, but MRI was not performed systematically or analyzed quantitatively. HLA-B27 status was available for all 160 included participants. The registry did not systematically capture the specific ASAS classification pathway fulfilled by each patient, namely imaging arm versus clinical arm, or structured MRI lesion-level findings. Therefore, imaging-arm and clinical-arm counts, as well as the frequency of supportive MRI findings among patients with non-radiographic axSpA, could not be reliably reconstructed from the locked de-identified analytic extract. Alternative diagnoses were excluded through routine rheumatologic assessment using clinical, laboratory, and imaging information. All patients recorded in the institutional axSpA registry during the study period were screened for eligibility. Of 220 patients assessed, 52 did not fulfill the ASAS classification criteria and 8 had incomplete baseline data, leaving 160 patients for analysis (Figure S1). Of the 8 patients excluded for incomplete baseline data, the missing information involved one or more of the baseline disease activity or covariate fields required for the primary analyses. Inclusion was based on eligibility within the institutional axSpA registry and the availability of complete baseline data, rather than on the initiation of biologic or targeted synthetic therapy. The cohort therefore included a mix of patients initiating or switching advanced therapy and patients who were not starting a new advanced-treatment course at baseline.

### 2.3. Variables and Measurements

For patients initiating or switching treatment, baseline was defined as the assessment at the start of the index medication episode, from which subsequent treatment outcomes were evaluated. For patients without an advanced-treatment initiation episode, baseline was defined as the first eligible registry assessment within the study period with complete disease activity and covariate data. Each patient contributed one baseline assessment only and appeared once in the analysis. Baseline data included age, sex, age at symptom onset and diagnosis, disease subtype (radiographic vs. non-radiographic axSpA), HLA-B27 status, smoking status, body mass index (BMI), and comorbidities, including fibromyalgia, psoriasis, inflammatory bowel disease, uveitis, hypertension, dyslipidemia, and osteoarthritis. All participants were clinically assessed for fibromyalgia at the baseline evaluation by the treating rheumatologist. The assessment was informed by the American College of Rheumatology (ACR) 2010/2016 criteria [17,18]; however, the Widespread Pain Index and Symptom Severity Scale were not recorded systematically for every participant. Fibromyalgia status was based on the rheumatologist-documented diagnosis present at baseline. Whether the diagnosing rheumatologist reviewed the BASDAI score before documenting fibromyalgia could not be determined retrospectively. Erythrocyte sedimentation rate (ESR) and C-reactive protein (CRP) were obtained at the baseline assessment, concurrently with the patient-reported measures, and were recorded as continuous values and categorized as elevated or normal. CRP was categorized relative to the institutional laboratory reference range (elevated CRP > 6 mg/L). ESR was analyzed primarily as a continuous variable; elevated ESR was defined using age- and sex-adjusted upper limits of normal, calculated as age/2 for men and (age + 10)/2 for women. Disease activity was assessed using the ASDAS-CRP and BASDAI (total score and its six component items, each 0–10); physical function was assessed using the Bath Ankylosing Spondylitis Functional Index (BASFI). BASDAI and BASFI were administered in Arabic or English according to patient preference during routine clinical care. The registry did not capture the language used for each individual assessment, and the validation status of the specific questionnaire versions could not be verified retrospectively. Diagnostic delay was defined as the interval between the age at symptom onset and the age at diagnosis. Disease duration was defined as the interval from symptom onset to the baseline assessment. Treatment exposure was recorded as the number of biologic or targeted synthetic agents and drug classes received.

The Ankylosing Spondylitis Disease Activity Score using C-reactive protein (ASDAS-CRP) was treated as a composite disease activity index combining patient-reported domains—back pain, peripheral pain or swelling, duration of morning stiffness, and patient global assessment—with CRP. It was therefore not interpreted as a purely objective or exclusively inflammatory measure. Because fibromyalgia and the pain-, fatigue-, and symptom-severity-weighted components of the Bath Ankylosing Spondylitis Disease Activity Index (BASDAI) assess overlapping experiences, adjustment for concomitant fibromyalgia may partly reflect shared symptom content. This potential circularity was considered when interpreting attenuation of the association between sex and BASDAI.

### 2.4. Statistical Analysis

Continuous variables were summarized as mean with standard deviation or median with interquartile range, according to their distribution, and categorical variables as number and percentage. Between-sex comparisons used the Welch t test for approximately normally distributed continuous variables, the Mann–Whitney U test for non-normally distributed variables, and the chi-square or Fisher exact test for categorical variables, as appropriate. Effect estimates are reported with 95% confidence intervals. For continuous outcomes, Cohen’s *d* was calculated from the untransformed values as the difference in group means divided by the pooled standard deviation. Cohen’s *d* was reported as a descriptive standardized effect size to facilitate comparison across outcomes and with prior literature. For non-normally distributed variables summarized using medians and interquartile ranges, statistical inference was based on the Mann–Whitney U test; Cohen’s *d* was not used as the basis for hypothesis testing.

BASDAI was defined as the primary outcome. The association between female sex and BASDAI was evaluated using sequential multivariable linear regression models. Model 1 included sex only. Model 2 additionally included age, disease duration, and radiographic subtype. Model 3 additionally included body mass index (BMI), and Model 4 additionally included concomitant fibromyalgia. Model 4 was defined as the primary adjusted model. Covariates were selected on clinical and conceptual grounds, informed by prior literature and the study objectives, rather than by univariable statistical significance or automated variable-selection procedures. Age, disease duration, and radiographic subtype were treated as potential confounders; BMI and concomitant fibromyalgia were added sequentially as clinically relevant factors of interest. The revised model hierarchy was defined before fitting the sequential models but was not included in a preregistered statistical analysis plan. ASDAS-CRP was deliberately excluded from the primary BASDAI models because the two indices share patient-reported symptom domains, and adjustment for ASDAS-CRP could introduce overadjustment and complicate interpretation. Change in the female-sex coefficient across the sequential models was interpreted as statistical attenuation and not as evidence of a causal or explanatory relationship. Analyses used complete cases, and no imputation was performed. Because complete baseline data were required for inclusion, all 160 participants contributed to the primary analyses; the analysis restricted to participants without fibromyalgia included 136 participants. Because the degree of attenuation in sequential models can depend on the order in which covariates are introduced, these models were not intended to partition the association into unique proportions attributable to BMI, fibromyalgia, or other covariates.

The Bath Ankylosing Spondylitis Functional Index (BASFI) and ASDAS-CRP were secondary outcomes and were examined using analogous sequential regression models. Erythrocyte sedimentation rate (ESR) was considered an exploratory outcome and was analyzed primarily as a continuous variable. Sensitivity analyses included restriction to participants without fibromyalgia, inclusion of a sex-by-fibromyalgia interaction term, and repetition of the ESR model using log-transformed ESR and heteroscedasticity-robust standard errors. The restricted analysis was interpreted cautiously because exclusion of patients with fibromyalgia reduced precision and changed the target population.

Benjamini–Hochberg false-discovery rate (FDR) correction was applied separately to the predefined families of descriptive baseline comparisons and disease activity/item-level comparisons. BASDAI was the primary outcome, BASFI and ASDAS-CRP were secondary outcomes, and ESR and item-level analyses were exploratory. The multivariable and sensitivity analyses were not included in the false-discovery rate correction and were interpreted as exploratory; the possibility of chance findings arising from multiple analyses was acknowledged.

Model results are reported as regression coefficients, 95% confidence intervals, *p* values, the number of participants included, and model-fit statistics. Collinearity was assessed using variance inflation factors, and residual distributions, linearity, and heteroscedasticity were examined to assess model assumptions. Analyses were performed using Python version 3.12.3 and statsmodels version 0.14.6. Linear regression models were fitted using statsmodels.formula.api.ols and the fit() method. Heteroscedasticity-robust sensitivity analyses used HC3 covariance estimates through fit(cov_type=“HC3”).

During the preparation of this manuscript, the authors used ChatGPT (OpenAI; GPT-5.5 Thinking and GPT-5.6 Thinking) for language editing and formatting assistance. The authors reviewed and edited the output and take full responsibility for the content of the manuscript.

## 3. Results

### 3.1. Cohort and Sex-Stratified Characteristics

Of the 160 patients, 56 (35.0%) were women and 104 (65.0%) were men (Table 1). After Benjamini–Hochberg correction, women had a higher prevalence of non-radiographic disease (62.5% vs. 33.7%), higher baseline BMI (29.7 vs. 26.0 kg/m^2^), more comorbid fibromyalgia (28.6% vs. 7.7%), and more frequently elevated ESR (53.6% vs. 29.8%) (all *q* < 0.05). HLA-B27 positivity was lower in women than in men, but this difference was not significant after correction (51.8% vs. 68.3%; *q* = 0.22). Age, age at onset, diagnostic delay, disease duration, smoking, and the remaining comorbidities did not differ significantly according to sex. Although fibromyalgia was more common in women, the overall comorbidity burden did not differ significantly between sexes (mean 1.8 vs. 1.4 recorded comorbidities; *p* = 0.10), and after excluding fibromyalgia, the groups were similar (1.5 vs. 1.4; *p* = 0.44), indicating that the female comorbidity excess was specific to fibromyalgia.

### 3.2. Disease Activity and Function: Divergence Between Symptom-Based and CRP-Anchored Measures

**Figure 1:**
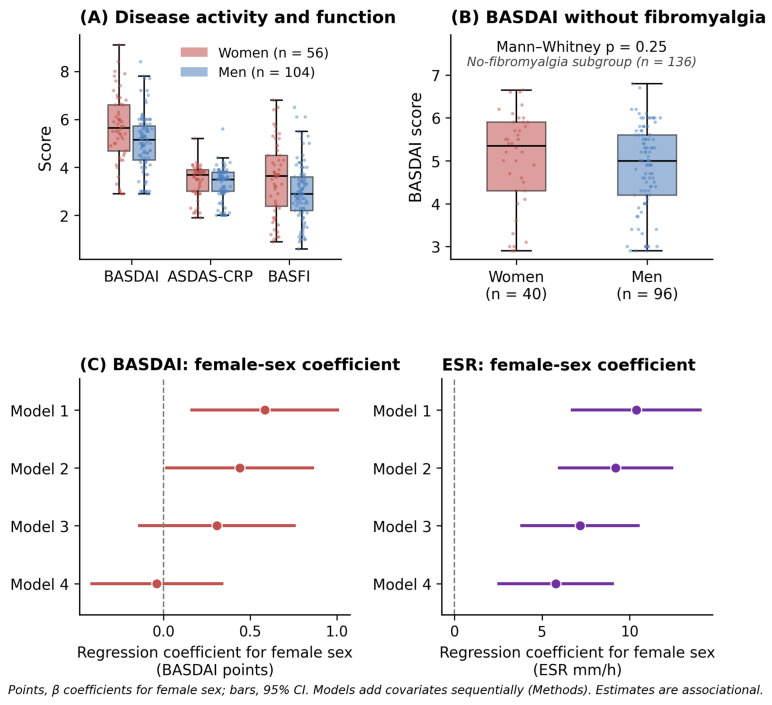
Sex differences in disease activity measures in axial spondyloarthritis. (**A**) Baseline BASDAI, ASDAS-CRP, and BASFI distributions by sex. (**B**) BASDAI distributions by sex in the sensitivity analysis restricted to participants without concomitant fibromyalgia. (**C**) Female-sex regression coefficients with 95% confidence intervals across sequentially adjusted models for BASDAI and ESR. Model 1 included sex only; Model 2 additionally included age, disease duration, and radiographic subtype; Model 3 additionally included BMI; and Model 4 additionally included concomitant fibromyalgia. Coefficient attenuation across models represents statistical adjustment and does not establish causal or explanatory relationships. In panel B, the reported *p* value is from the Mann–Whitney U test.

Disease activity diverged according to the type of measure used (Table 2; Figure 1A). Women scored significantly higher on the symptom-based BASDAI than men (median 5.65 [IQR 4.67–6.60] vs. 5.15 [4.30–5.72]; *p* = 0.007). The difference in BASFI scores was nominally significant before multiple testing correction but borderline afterwards (median 3.65 [IQR 2.38–4.50] vs. 2.90 [2.20–3.60]; *p* = 0.023, *q* = 0.052). The CRP-anchored ASDAS-CRP was numerically higher in women but did not differ significantly (median 3.70 [IQR 3.00–3.90] vs. 3.50 [3.00–3.80]; *p* = 0.099). Item-level analysis localized the female BASDAI excess to the fatigue and spinal-pain items (*q* = 0.044 for both), with a non-significant trend for the peripheral-pain item and no sex difference in the enthesitis or morning stiffness items. The standardized between-sex differences were small to moderate for BASDAI (Cohen’s *d* = 0.46, 95% CI 0.13 to 0.78) and BASFI (d = 0.39, 95% CI 0.06 to 0.72), small and imprecise for ASDAS-CRP (d = 0.18, 95% CI −0.14 to 0.51), and large for ESR (d = 0.93, 95% CI 0.59 to 1.27).

### 3.3. Sequential Adjustment of Associations Between Sex and Study Outcomes

We evaluated the association of female sex with each outcome using sequential multivariable linear regression models (Table 3; Figure 1C). In the unadjusted model, female sex was associated with a higher BASDAI score (β = 0.58, 95% CI 0.16 to 1.00; *p* = 0.007). The female-sex coefficient was progressively attenuated after adjustment for age, disease duration, and radiographic subtype (β = 0.44, 95% CI 0.02 to 0.86; *p* = 0.040), and after the further addition of BMI (β = 0.31, 95% CI −0.14 to 0.75; *p* = 0.175). Further attenuation occurred after concomitant fibromyalgia was added to the model, at which point the female-sex coefficient was close to zero and not statistically significant (β = −0.04, 95% CI −0.41 to 0.34; *p* = 0.843). In the fully adjusted model, concomitant fibromyalgia was associated with a higher BASDAI score (β = 2.04, 95% CI 1.58 to 2.51; *p* < 0.001), and the adjusted R^2^ increased from 0.04 in Model 1 to 0.42 in Model 4.

BASFI showed a similar attenuation pattern: the female-sex coefficient was β = 0.52 (95% CI 0.08 to 0.95; *p* = 0.021) in Model 1 and β = 0.03 (95% CI −0.41 to 0.48; *p* = 0.878) in Model 4. Female sex was not statistically significantly associated with ASDAS-CRP in any model; in Model 4, the coefficient was β = −0.11 (95% CI −0.34 to 0.13; *p* = 0.367). For ESR, the association with female sex was reduced across the sequential models but remained statistically significant after full adjustment (β = 5.77 mm/h, 95% CI 2.54 to 9.00; *p* < 0.001).

The higher ESR values observed in women were not paralleled by continuous CRP [median 3.5 (IQR 1.6–6.5) versus 3.0 (IQR 1.3–5.0) mg/L; *p* = 0.250] or elevated CRP status (26.8% versus 17.3%; *p* = 0.227), whereas elevated ESR with normal CRP was more frequent in women (29% versus 14%; *p* = 0.037). Variance inflation factors were below 3.1. The primary BASDAI model showed no evidence of heteroscedasticity (Breusch–Pagan *p* = 0.420). Residuals showed a mild departure from normality (Shapiro–Wilk *p* = 0.0012), but the female-sex estimate and inference were unchanged when heteroscedasticity-robust standard errors were used.

### 3.4. Sensitivity Analyses

In the analysis restricted to participants without fibromyalgia (*n* = 136), and after adjustment for age, disease duration, radiographic subtype, and BMI, the estimated female-sex coefficients were small and imprecise for BASDAI (β = −0.06, 95% CI −0.48 to 0.36; *p* = 0.781; Figure 1B), BASFI (β = −0.07, 95% CI −0.55 to 0.40; *p* = 0.761), and ASDAS-CRP (β = −0.14, 95% CI −0.40 to 0.13; *p* = 0.300). The association with ESR remained statistically significant (β = 4.35 mm/h, 95% CI 0.74 to 7.95; *p* = 0.019; Table S1). The standardized BASDAI difference decreased from Cohen’s *d* = 0.46 in the full cohort to d = 0.18 after restricting the analysis to participants without fibromyalgia. This restricted analysis should be interpreted cautiously because it reduced sample size and changed the target population.

In the full cohort, there was no statistical evidence of a sex-by-fibromyalgia interaction for BASDAI (interaction β = −0.15, 95% CI −1.12 to 0.82; *p* = 0.759; Table S1), indicating no evidence that the association between sex and BASDAI differed according to fibromyalgia status. The corresponding interaction analyses for BASFI, ASDAS-CRP, and ESR are also reported in Table S1.

### 3.5. Findings That Did Not Differ by Sex

HLA-B27 positivity and diagnostic delay did not differ significantly by sex; diagnostic delay was numerically longer in women (5.0 vs. 4.1 years) but non-significant after adjustment for age at onset, subtype, and HLA-B27 (β = 0.54 years, *p* = 0.33).

The cohort was not restricted to patients initiating biologic or targeted synthetic therapy. Available registry information indicated that participants included both patients initiating or switching advanced therapy and patients not starting a new advanced-treatment course at baseline. Structured baseline medication details by sex, including specific drug class, dose, treatment duration, adherence, and concomitant analgesic or psychotropic medication use, were not systematically available in the locked analytic extract.

As a separate, exploratory retrospective longitudinal component, twelve-month outcomes and cumulative biologic or targeted synthetic treatment exposure are summarized descriptively in Supplementary Table S2.

## 4. Discussion

In this single-center Saudi cohort, women with axSpA differed from men in several respects, including more non-radiographic disease, higher BMI, more comorbid fibromyalgia, and more frequently elevated ESR. Differences in disease activity and function were concentrated in patient-reported outcomes: women scored higher on the symptom-based BASDAI and, to a borderline degree, on the patient-reported BASFI, whereas the CRP-anchored ASDAS-CRP did not differ significantly by sex. The female BASDAI excess was localized to the fatigue and spinal-pain items. In exploratory cross-sectional models, the female-sex coefficient for BASDAI was attenuated after adjustment for BMI and concomitant fibromyalgia; however, this attenuation should be interpreted as statistical attenuation only and not as evidence that fibromyalgia explains the observed sex difference. These cross-sectional associations do not establish temporal ordering or causation. In the analysis restricted to participants without fibromyalgia, the estimated sex differences in BASDAI, ASDAS-CRP, and BASFI were smaller and imprecise, but this analysis had reduced precision and changed the target population.

Our findings are consistent with the international literature, in which higher patient-reported activity in women alongside CRP-anchored composite activity that does not differ significantly by sex has been reported in larger cohorts and meta-analyses [7,19,20]. The dissociation we observed—higher BASDAI scores in women but no significant sex difference in ASDAS-CRP—is the central finding of the systematic review and meta-analysis of disease activity indices by sex, where the pooled BASDAI difference favored women but ASDAS-CRP showed none [7]. Our BASDAI gap is close to that pooled estimate, and large registries report the same pattern: patient-reported activity differs by sex, but CRP-anchored activity does not [19,20]. Although statistically significant, the absolute between-sex BASDAI difference was approximately half a point on the 0–10 scale and modest in magnitude (Cohen’s *d* = 0.46). A formal anchor-based minimal clinically important difference was not derived in this dataset; therefore, the observed difference should not be interpreted in isolation as sufficient to guide treatment decisions. It is well established that fibromyalgia inflates patient-reported activity and that obesity raises inflammatory markers [8,9,10,11,12,13]. The present study jointly evaluates these factors and quantifies their associations within a single cohort. The excess of non-radiographic disease in our women fits earlier real-life cohorts [21], as do sex differences in physical function [22].

The attenuation of the female-sex coefficient after adding concomitant fibromyalgia should be interpreted cautiously. Fibromyalgia and BASDAI share symptom domains, particularly pain, fatigue, and symptom severity, and the Widespread Pain Index and Symptom Severity Scale were not systematically recorded. Therefore, the observed attenuation may partly reflect overlapping symptom content, clinical classification overlap, or possible circularity rather than an independent explanatory effect of fibromyalgia. The findings support an association between documented concomitant fibromyalgia and higher symptom-reported disease activity, but they do not establish that fibromyalgia causes, mediates, or fully explains the observed sex difference. Alternative biological, psychosocial, cultural, treatment-related, and measurement-related explanations cannot be excluded. Fibromyalgia-related symptom burden may reflect interacting biological, psychological, and social factors [23], although these mechanisms were not evaluated in the present study. Interrelationships among disease activity, central sensitization, psychosocial characteristics, and lifestyle factors have also been described in axSpA [24].

Women had higher ESR values, whereas CRP and ASDAS-CRP did not differ significantly by sex. Several factors may contribute to ESR variation, including age, sex, adiposity, anemia, renal function, immunoglobulin concentrations, infection, treatment exposure, and other comorbidities. BMI was associated with part of the observed ESR difference, but the cause of the higher ESR in women cannot be established from the available data. The absence of a corresponding difference in CRP supports cautious interpretation but does not exclude inflammatory activity that was not captured by CRP. ESR should therefore be interpreted in conjunction with CRP, symptoms, clinical examination, relevant comorbidities, and imaging when clinically indicated. Because contemporaneous imaging-based measures of axial inflammation and several other determinants of ESR were unavailable, the present study cannot determine whether the observed ESR difference was inflammatory, non-inflammatory, or multifactorial.

From a methodological perspective, the sequentially adjusted models were selected to provide a transparent assessment of how the association between sex and each outcome changed after accounting for clinically relevant covariates. This approach was preferred over a formal causal-modeling framework because fibromyalgia status, BMI, and the disease activity outcomes were assessed cross-sectionally at the same baseline visit, preventing verification of temporal ordering or causal assumptions. Changes in the female-sex coefficient across models are therefore interpreted as statistical attenuation only. They do not establish that any covariate causes or fully explains the observed sex difference.

These findings may have implications for the interpretation of disease activity measures in clinical practice. When symptom-reported scores are high, concomitant fibromyalgia and other non-inflammatory contributors should be considered alongside clinical assessment, inflammatory markers, and composite indices incorporating CRP. However, this study did not directly evaluate treatment decisions, overtreatment, inappropriate treatment escalation, differential treatment response, imaging-defined inflammation, or the clinical consequences of selecting BASDAI rather than ASDAS-CRP to guide therapy. Any effect of concomitant fibromyalgia on treatment escalation should therefore be regarded as a hypothesis requiring prospective evaluation. Symptoms such as pain, fatigue, and functional limitation remain clinically important regardless of their underlying mechanism and require appropriate assessment and management.

Prospective longitudinal studies with repeated assessment of patient-reported outcomes, CRP, ESR, imaging, treatment exposure, physical activity, sleep, and psychosocial factors are needed to clarify the temporal relationships among inflammatory activity, fibromyalgia-related symptom burden, and sex. Interventional studies should also determine whether targeted management of fibromyalgia-related symptoms, sleep disturbance, physical inactivity, and excess weight modifies symptom burden and functional outcomes independently of inflammatory disease control.

### Limitations

This study has several limitations. First, the principal analysis was cross-sectional; therefore, the temporal relationships among sex, concomitant fibromyalgia, BMI, and disease activity measures could not be established. Attenuation of the female-sex coefficient after covariate adjustment should not be interpreted as evidence that fibromyalgia causes or explains the observed difference. In addition, the magnitude of attenuation across sequential models depends partly on the order of covariate entry. Because BMI was entered before concomitant fibromyalgia, the change in the female-sex coefficient after adding fibromyalgia should not be interpreted as the amount uniquely attributable to fibromyalgia, and the models do not quantify the proportion of the sex association explained by each variable.

Second, the study was conducted using an institutional registry at a single tertiary center. Referral patterns, treatment selection, healthcare access, and the characteristics of patients managed at a tertiary center may introduce selection bias and limit generalizability. The prevalence and recognition of fibromyalgia, BMI distribution, prevalence of obesity, HLA-B27 frequency, lifestyle, physical activity, climate, and sociocultural influences on symptom reporting may also differ across populations [25]. The findings should therefore be extrapolated cautiously beyond similar clinical settings. In addition, because inclusion required complete baseline data, the primary analyses were restricted to complete cases; a detailed comparison of the 8 excluded patients with those included was limited by their incomplete baseline information, and complete-case inclusion may therefore have introduced selection bias.

Third, although all participants were clinically assessed for fibromyalgia, the assessment was not supported by systematically recorded Widespread Pain Index and Symptom Severity Scale scores. Variation in clinical documentation and possible misclassification therefore cannot be excluded. In addition, fibromyalgia and several BASDAI components assess overlapping domains, particularly pain, fatigue, and symptom severity. This shared symptom content may partly account for the observed association and introduces potential circularity when fibromyalgia is included as a covariate.

Fourth, ASDAS-CRP combines patient-reported domains with CRP and should not be interpreted as a purely objective measure of inflammation. The absence of a statistically significant sex difference in ASDAS-CRP or CRP does not establish equivalence and does not exclude differences in inflammatory activity that were not captured by these measures. Contemporaneous imaging-based measures of axial inflammation were not included in the analysis. ASAS imaging-arm and clinical-arm classification counts and structured MRI lesion data were not systematically available. Non-radiographic axSpA classification therefore relied on routine rheumatology documentation, HLA-B27 testing, pelvic radiograph reports, and clinically indicated MRI where available, rather than on central imaging rereading or standardized MRI scoring.

Fifth, treatment exposure and several potential confounders were not fully characterized or incorporated into the primary models. Detailed medication type, dose, duration, adherence, concomitant analgesic or psychotropic medication use, physical activity, sleep quality, socioeconomic factors, and psychosocial stressors were not systematically available. The distribution of advanced therapies and whether treatment patterns differed by sex were therefore not characterized. Residual confounding and confounding by indication therefore remain possible.

Sixth, ESR is affected by multiple inflammatory and non-inflammatory determinants. Information on several additional determinants of ESR, including anemia, renal function, immunoglobulin concentrations, infection, treatment exposure, and other comorbidities, was not available in the registry dataset; these variables were not incorporated into the analysis. The cause of the higher ESR observed in women therefore cannot be established.

Finally, the sample size was modest, and only 24 participants had fibromyalgia. Models containing fibromyalgia may consequently have limited precision and unstable estimates, particularly in subgroup and interaction analyses. The number of comparisons and exploratory models also increases the possibility of chance findings. These results should therefore be interpreted cautiously and confirmed in larger prospective multicenter studies using systematic fibromyalgia assessment, comprehensive confounder measurement, and imaging-based measures of inflammation.

## 5. Conclusions

In this retrospective cross-sectional analysis of a Saudi axSpA cohort, women had higher BASDAI scores than men, whereas no statistically significant sex difference was observed in ASDAS-CRP. The association between female sex and BASDAI was progressively attenuated after sequential adjustment for clinical covariates, including BMI and concomitant fibromyalgia. However, this attenuation should be interpreted as a statistical attenuation only and may partly reflect overlapping symptom content and possible classification circularity between fibromyalgia and BASDAI, rather than evidence that fibromyalgia explains the observed sex difference. The cross-sectional design, overlap between fibromyalgia symptoms and BASDAI components, limited number of patients with fibromyalgia, and absence of imaging-based measures of inflammation require cautious interpretation. Prospective studies using systematic fibromyalgia assessment, repeated disease activity measurements, comprehensive confounder evaluation, and objective imaging measures are needed to confirm these findings.

## Figures and Tables

**Table 1:** Baseline demographic, clinical, and comorbidity characteristics by sex.

Characteristic	Women (*n* = 56)	Men (*n* = 104)	*p*	*q* (FDR)
Age, years—mean (SD)	42.9 (11.7)	39.7 (12.9)	0.113	0.303
Age at symptom onset, years	30.2 (7.9)	28.8 (8.5)	0.285	0.428
Diagnostic delay, years	5.0 (3.7)	4.1 (3.0)	0.118	0.303
Disease duration, years	12.7 (7.4)	10.9 (6.6)	0.137	0.308
BMI, kg/m^2^	29.7 (5.1)	26.0 (4.4)	<0.001	<0.001
Non-radiographic axSpA—*n* (%)	35 (62.5)	35 (33.7)	0.001	0.006
HLA-B27 positive	29 (51.8)	71 (68.3)	0.060	0.215
Current smoker	9 (16.1)	27 (26.0)	0.219	0.371
Fibromyalgia	16 (28.6)	8 (7.7)	0.001	0.006
Psoriasis	13 (23.2)	14 (13.5)	0.177	0.354
Inflammatory bowel disease	7 (12.5)	8 (7.7)	0.477	0.573
Uveitis	8 (14.3)	23 (22.1)	0.324	0.441
Hypertension	8 (14.3)	15 (14.4)	1.000	1.000
High cholesterol	16 (28.6)	27 (26.0)	0.866	0.917
High triglycerides	15 (26.8)	24 (23.1)	0.743	0.836
Osteoarthritis	9 (16.1)	10 (9.6)	0.343	0.441
CRP, mg/L—median (IQR)	3.5 (1.6–6.5)	3.0 (1.3–5.0)	0.250	0.385
ESR, mm/h—median (IQR)	24 (15–37)	15 (10–22)	<0.001	<0.001
Elevated ESR	30 (53.6)	31 (29.8)	0.005	0.024
Elevated CRP (>6 mg/L)	15 (26.8)	18 (17.3)	0.227	0.371

Note. Continuous variables are shown as mean (SD) unless otherwise indicated and were compared by Welch t test or Mann–Whitney U test, as appropriate; categorical variables are *n* (%), compared by χ^2^/Fisher exact. *q* = Benjamini–Hochberg false discovery rate-adjusted *p*-value. ESR and CRP were analyzed as continuous values and as elevated/normal status; elevated ESR was defined using age- and sex-adjusted upper limits of normal, calculated as age/2 for men and (age + 10)/2 for women; elevated CRP was defined as >6 mg/L. Abbreviations: axSpA, axial spondyloarthritis; BMI, body mass index; CRP, C-reactive protein; ESR, erythrocyte sedimentation rate; FDR, false discovery rate; HLA-B27, human leukocyte antigen B27; IQR, interquartile range; SD, standard deviation.

**Table 2:** Disease activity and physical function by sex, with BASDAI item-level breakdown.

Measure—Median (IQR)	Women (*n* = 56)	Men (*n* = 104)	*p*	*q* (FDR)	Cohen’s *d* (95% CI)
ASDAS-CRP (CRP-anchored composite)	3.70 (3.00–3.90)	3.50 (3.00–3.80)	0.099	0.148	0.18 (−0.14 to 0.51)
BASDAI total (patient-reported)	5.65 (4.67–6.60)	5.15 (4.30–5.72)	0.007	0.044	0.46 (0.13 to 0.78)
BASFI (function)	3.65 (2.38–4.50)	2.90 (2.20–3.60)	0.023	0.052	0.39 (0.06 to 0.72)
BASDAI item—fatigue	4.00 (3.00–8.00)	4.00 (3.00–4.00)	0.010	0.044	0.58 (0.25 to 0.91)
BASDAI item—spinal pain	4.00 (3.00–8.00)	3.50 (3.00–5.00)	0.015	0.044	0.53 (0.20 to 0.86)
BASDAI item—peripheral pain	6.00 (5.00–7.00)	6.00 (5.00–7.00)	0.071	0.128	0.31 (−0.01 to 0.64)
BASDAI item—enthesitis	6.00 (5.00–7.00)	6.00 (5.00–7.00)	0.966	0.966	−0.01 (−0.34 to 0.31)
BASDAI item—stiffness severity	6.00 (5.00–7.00)	6.00 (5.00–7.00)	0.919	0.966	0.01 (−0.31 to 0.34)
BASDAI item—stiffness duration	6.00 (5.00–7.00)	6.00 (5.00–7.00)	0.700	0.901	−0.02 (−0.34 to 0.31)

Note. Values are median (interquartile range). Disease activity scores and BASDAI items (indented) were compared using the Mann–Whitney U test. *d* = Cohen’s *d* for women versus men, calculated from untransformed continuous values as the difference in group means divided by the pooled standard deviation and shown with its 95% confidence interval. For variables summarized as median (interquartile range), *d* is presented as a descriptive standardized effect size only; statistical inference was based on the Mann–Whitney U test. Abbreviations: ASDAS-CRP, Ankylosing Spondylitis Disease Activity Score using C-reactive protein; BASDAI, Bath Ankylosing Spondylitis Disease Activity Index; BASFI, Bath Ankylosing Spondylitis Functional Index; CI, confidence interval; FDR, false discovery rate; IQR, interquartile range.

**Table 3:** Sequential multivariable linear regression models evaluating the association between female sex and disease activity, functional, and inflammatory-marker outcomes.

**Panel A. Female-sex coefficient across sequentially adjusted models.**							
**Outcome**	**Model**	**Adjustment set**	**β (female sex)**	**95% CI**	***p***	***n***	**Adj. R^2^**
BASDAI	Model 1	Sex only	0.58	0.16 to 1.00	0.007	160	0.04
	Model 2	+ age, disease duration, radiographic subtype	0.44	0.02 to 0.86	0.040	160	0.13
	Model 3	+ BMI	0.31	−0.14 to 0.75	0.175	160	0.14
	Model 4	+ concomitant fibromyalgia	−0.04	−0.41 to 0.34	0.843	160	0.42
BASFI	Model 1	Sex only	0.52	0.08 to 0.95	0.021	160	0.03
	Model 2	+ age, disease duration, radiographic subtype	0.31	−0.11 to 0.73	0.147	160	0.18
	Model 3	+ BMI	0.21	−0.24 to 0.66	0.350	160	0.18
	Model 4	+ concomitant fibromyalgia	0.03	−0.41 to 0.48	0.878	160	0.25
ASDAS-CRP	Model 1	Sex only	0.12	−0.10 to 0.34	0.271	160	0.00
	Model 2	+ age, disease duration, radiographic subtype	0.05	−0.17 to 0.27	0.652	160	0.07
	Model 3	+ BMI	−0.03	−0.26 to 0.21	0.815	160	0.08
	Model 4	+ concomitant fibromyalgia	−0.11	−0.34 to 0.13	0.367	160	0.13
ESR (mm/h)	Model 1	Sex only	10.37	6.73 to 14.02	<0.001	160	0.16
	Model 2	+ age, disease duration, radiographic subtype	9.19	5.98 to 12.40	<0.001	160	0.41
	Model 3	+ BMI	7.17	3.85 to 10.48	<0.001	160	0.45
	Model 4	+ concomitant fibromyalgia	5.77	2.54 to 9.00	<0.001	160	0.50
**Panel B. Complete coefficients of the primary BASDAI model (Model 4).**							
**Term**			**β**		**95% CI**		***p***
Female sex			−0.038		−0.412 to 0.337		0.843
Age (per year)			0.019		−0.003 to 0.040		0.095
Disease duration (per year)			0.003		−0.035 to 0.041		0.870
Non-radiographic subtype			0.072		−0.264 to 0.407		0.673
BMI (per kg/m^2^)			0.029		−0.009 to 0.066		0.131
Concomitant fibromyalgia			2.044		1.580 to 2.509		<0.001

Note. β denotes the unstandardized regression coefficient. In Panel A, β represents the coefficient for female sex, with male sex as the reference category. Models were fitted sequentially: Model 1 included sex only; Model 2 additionally included age, disease duration, and radiographic subtype; Model 3 additionally included body mass index (BMI); and Model 4 additionally included concomitant fibromyalgia. Outcomes were BASDAI (primary), BASFI, ASDAS-CRP, and ESR; *n* = 160 for all models. For the BASDAI models, R^2^ was 0.05, 0.15, 0.17, and 0.44 (adjusted R^2^ 0.04, 0.13, 0.14, and 0.42) for Models 1–4, respectively. Panel B presents the complete coefficients of the primary BASDAI model (Model 4). Attenuation of the female-sex coefficient across models reflects statistical adjustment and does not establish causal or explanatory relationships. Abbreviations: ASDAS-CRP, Ankylosing Spondylitis Disease Activity Score using C-reactive protein; BASDAI, Bath Ankylosing Spondylitis Disease Activity Index; BASFI, Bath Ankylosing Spondylitis Functional Index; BMI, body mass index; CI, confidence interval; ESR, erythrocyte sedimentation rate.

## Data Availability

The data presented in this study are available upon request from the corresponding author. The data are not publicly available owing to privacy and ethical restrictions.

## Supplementary Materials

The following supporting information can be downloaded at: https://www.mdpi.com/article/10.3390/jcm15145602/s1, Figure S1: Participant flow diagram; Table S1: Sensitivity and robustness analyses of the associations between female sex and study outcomes; Table S2: Exploratory 12-month outcomes and cumulative treatment exposure by sex.
