# Supplementary material for: Sex Differences in Disease Activity Measures in Axial Spondyloarthritis and Their Association with Concomitant Fibromyalgia: A Retrospective Cross-Sectional Analysis of a Saudi Cohort
Source: J Clin Med. 2026 Jul 17;15(14):5602. doi: 10.3390/jcm15145602 (PMC13413398; doi:10.3390/jcm15145602)
Supplement: Supplementary file 1 [file jcm-15-05602-s001.zip › Table_S1.pdf]

Table S1. Sensitivity and robustness analyses of the associations between female sex and study outcomes.

Panel A. Analysis restricted to participants without concomitant fibromyalgia (n = 136).

Outcome	β (female sex)	95% CI	p	n
BASDAI	−0.06	−0.48 to 0.36	0.781	136
BASFI	−0.07	−0.55 to 0.40	0.761	136
ASDAS-CRP	−0.14	−0.40 to 0.13	0.300	136
ESR (mm/h)	4.35	0.74 to 7.95	0.019	136

Each model adjusted for age, disease duration, radiographic subtype, and BMI. β denotes the unstandardized regression coefficient for female sex (reference category, male).

Panel B. Sex × fibromyalgia interaction in the full cohort (n = 160).

Outcome	Interaction β	95% CI	p	n
BASDAI	−0.15	−1.12 to 0.82	0.759	160
BASFI	0.48	−0.66 to 1.61	0.405	160
ASDAS-CRP	−0.01	−0.61 to 0.60	0.988	160
ESR (mm/h)	7.40	−0.85 to 15.66	0.078	160

Each model includes sex, fibromyalgia, the sex × fibromyalgia interaction, age, disease duration, radiographic subtype, and BMI. The interaction β is the additional female-sex effect among participants with fibromyalgia.

Panel C. Robustness analyses.

Analysis	β (female sex)	95% CI	p	n
Primary BASDAI Model 4, HC3 robust SE	−0.04	−0.41 to 0.34	0.845	160
ESR Model 4, log-transformed ESR, HC3 robust SE	0.30	0.14 to 0.46	<0.001	160

Primary BASDAI Model 4 adjusts for age, disease duration, radiographic subtype, BMI, and concomitant fibromyalgia. HC3 = heteroscedasticity-consistent (HC3) robust standard errors. The log-ESR coefficient is reported on the natural-log scale; exp(β) = 1.35. Abbreviations: ASDAS-CRP, Ankylosing Spondylitis Disease Activity Score using C-reactive protein; BASDAI, Bath Ankylosing Spondylitis Disease Activity Index; BASFI, Bath Ankylosing Spondylitis Functional Index; BMI, body mass index; CI, confidence interval; ESR, erythrocyte sedimentation rate; SE, standard error.
