# Supplementary material for: Sex Differences in Disease Activity Measures in Axial Spondyloarthritis and Their Association with Concomitant Fibromyalgia: A Retrospective Cross-Sectional Analysis of a Saudi Cohort
Source: J Clin Med. 2026 Jul 17;15(14):5602. doi: 10.3390/jcm15145602 (PMC13413398; doi:10.3390/jcm15145602)
Supplement: Supplementary file 1 [file jcm-15-05602-s001.zip › Table_S2.pdf]

## Table S2. Exploratory 12-month outcomes and cumulative treatment exposure by sex.

### Panel A. Exploratory 12-month outcomes.

| Measure | Women (n = 56) | Men (n = 104) |
|---|---|---|
| 12-month follow-up available, n (%) | 56 (100) | 104 (100) |
| BASDAI, baseline | 5.65 (4.67–6.60) | 5.15 (4.30–5.72) |
| BASDAI, 12 months | 3.15 (2.20–4.10) | 2.90 (2.20–4.00) |
| BASDAI, change from baseline | −2.40 (−2.93 to −1.67) | −1.85 (−2.60 to −1.20) |
| ASDAS-CRP, baseline | 3.70 (3.00–3.90) | 3.50 (3.00–3.80) |
| ASDAS-CRP, 12 months | 2.00 (1.50–2.60) | 2.00 (1.50–2.80) |
| ASDAS-CRP, change from baseline | −1.55 (−1.83 to −1.00) | −1.05 (−1.80 to −0.80) |
| BASFI, baseline | 3.65 (2.38–4.50) | 2.90 (2.20–3.60) |
| BASFI, 12 months | 1.30 (1.00–1.80) | 1.20 (0.88–1.50) |
| BASFI, change from baseline | −2.15 (−3.10 to −1.25) | −1.85 (−2.30 to −1.08) |

*Values are median (interquartile range) unless otherwise indicated. No data were missing for these measures (n = 56 women, 104 men). These exploratory, uncontrolled analyses are descriptive and were not formally compared between sexes or adjusted for treatment.*

### Panel B. Cumulative treatment exposure.

| Measure | Women (n = 56) | Men (n = 104) |
|---|---|---|
| Advanced agents received, median (IQR) | 2 (1–3) | 2 (1–2) |
| Therapeutic classes received, median (IQR) | 1 (1–2) | 1 (1–2) |
| ≥2 advanced agents, n (%) | 34 (60.7) | 53 (51.0) |
| ≥2 therapeutic classes, n (%) | 18 (32.1) | 29 (27.9) |

*Cumulative exposure over the registry follow-up period. No data were missing for these measures. Baseline drug-class distribution by sex could not be reconstructed and is not reported. Abbreviations: ASDAS-CRP, Ankylosing Spondylitis Disease Activity Score using C-reactive protein; BASDAI, Bath Ankylosing Spondylitis Disease Activity Index; BASFI, Bath Ankylosing Spondylitis Functional Index; IQR, interquartile range.*
