# Supplementary figures and images for: Sex Differences in Disease Activity Measures in Axial Spondyloarthritis and Their Association with Concomitant Fibromyalgia: A Retrospective Cross-Sectional Analysis of a Saudi Cohort
Source: J Clin Med. 2026 Jul 17;15(14):5602. doi: 10.3390/jcm15145602 (PMC13413398; doi:10.3390/jcm15145602)

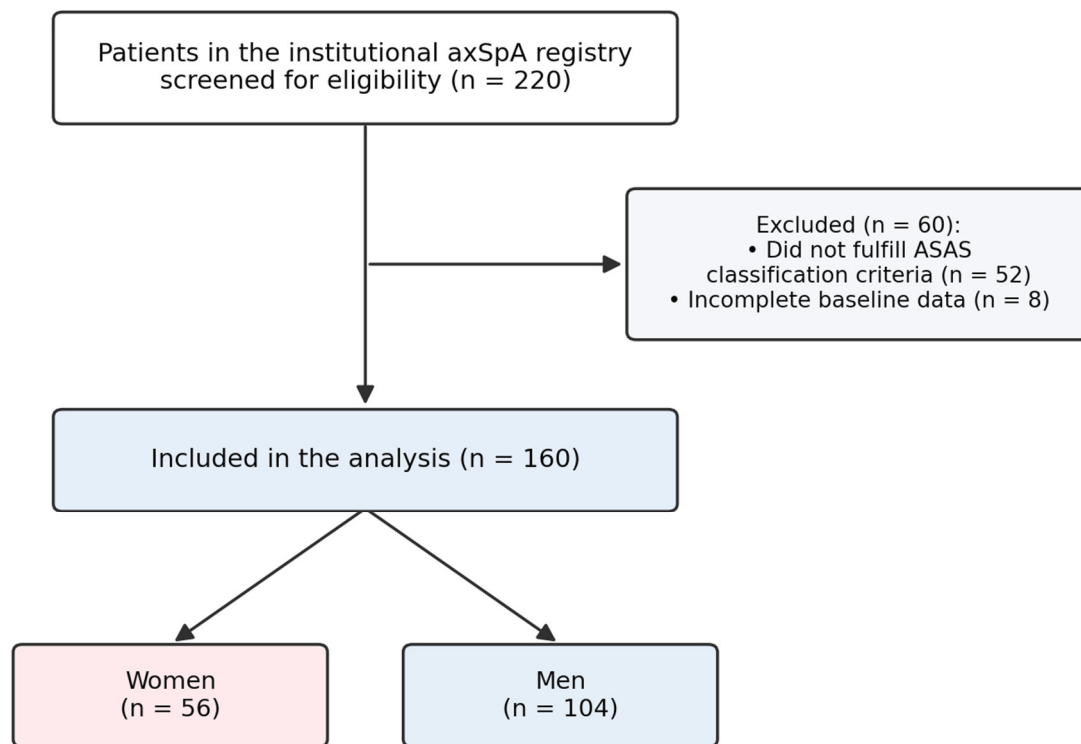

Figure S1: Participant flow diagram

Supplement: Supplementary file 1 [file jcm-15-05602-s001.zip › Figure S1.pdf]
